# Non-ulcerated necrotizing sialometaplasia may mimic a salivary gland tumor

**DOI:** 10.4322/acr.2021.244

**Published:** 2021-04-19

**Authors:** Patrícia Maria Fernandes, Erika Graf Pedroso, Alan Roger Santos-Silva, Pablo Agustin Vargas, Márcio Ajudarte Lopes

**Affiliations:** 1 Universidade Estadual de Campinas (UNICAMP), Faculdade de Odontologia de Piracicaba, Departamento de Diagnóstico Oral, Piracicaba, SP, Brasil

**Keywords:** Necrotizing sialometaplasia, salivary gland disease, head and neck neoplasm, oral pathology

## Abstract

Necrotizing sialometaplasia (NS) is a benign, self-limiting inflammatory entity that mainly affects the minor salivary glands located in the hard palate. Classically, NS is characterized as a nodule that evolves to a central ulcer. The most widely recognized triggering factor is an ischemic event. The diagnosis becomes a challenge in non-ulcerated NS cases which is essential to rule out the possibility of salivary gland tumors, especially the malignant ones. Here, we presented a case of a 32-year-old male patient with a 1-month complaint of a painful, slightly elevated erythematous area on the hard palate. Incisional biopsy was performed, and NS was diagnosed based on histopathological and immunohistochemical analyses. Clinicians should be aware of and consider NS as a differential diagnosis of minor salivary gland tumors, particularly when it presents as a non-ulcerated clinical aspect.

## INTRODUCTION

The hard palate harbors several minor salivary glands, which are the main structures affected by pathological changes in the palate. Among the most important alterations, inflammatory conditions and neoplasia are important to highlight. Necrotizing sialometaplasia (NS) is a well-described inflammatory disease that in most cases affects the minor salivary glands located in the hard palate. Classically, NS is characterized as a nodule that evolves to a central ulcer around 1 cm in diameter. Although an ischemic event is the most widely recognized triggering factor, local trauma, drug abuse and eating disorders are also possible causes.^1^ ^-^ ^3^

It is important to emphasize that the clinical features of ulcerated NS may be similar to malignant salivary gland tumors and, histologically, NS may mimic mucoepidermoid carcinoma, the most common malignant salivary gland tumor.^4^ Occasionally, NS may clinically present as a non-ulcerated aspect. In this scenario, where only a bluish red swelling is observed, differential diagnosis of malignant salivary gland tumors should also be considered. However, there are only few reports in the English language literature of non-ulcerated NS without a clear narrative of an ischemic event.^1^ ^,^ ^5^ ^-^ ^12^ Therefore, the aim of this report was to describe the clinical and histopathological features of a case of non-ulcerated NS and highlight the importance of considering malignant salivary gland tumor as a differential diagnosis.

## CASE REPORT

A 32-year-old male Caucasian patient was referred for evaluation of an oral lesion with about 1 month of evolution. His medical history was unremarkable, although he reported smoking, drinking and cocaine abuse.

**Figure 1:**
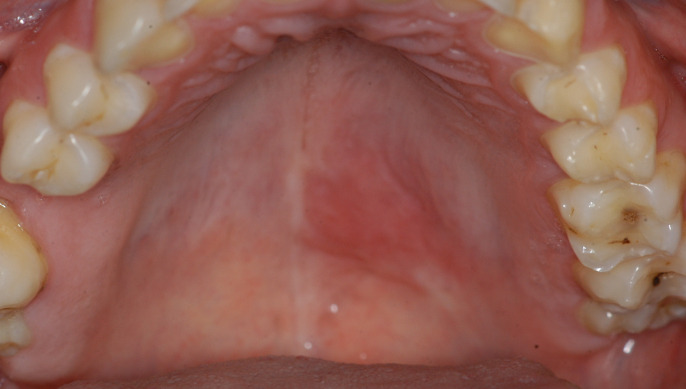
Clinical appearance of a non-ulcerated lesion showing a discreet elevated and erythematous area on the left hard palate.

Intraoral clinical examination showed a slightly elevated area on the hard palate measuring about 1 cm in diameter, which was firm and painful on palpation and covered by erythematous mucosa (Figure 1). In addition, the patient reported that he had been taking sodium diclofenac for about 10 days without improvement.

Based on the history of the chief complaint and clinical examination, the patient was submitted to incisional biopsy under local anesthesia. The histopathological analysis revealed a fragment of oral mucosa covered by stratified epithelium without ulceration, and salivary ducts with squamous metaplasia were observed in subjacent connective tissue (Figures 22B). The immunohistochemical study showed strong positivity to CK7 in the ductal epithelium (Figure 22D) and also to CK14 in the areas of squamous metaplasia (Figures 33B). Therefore, these microscopic features were compatible with a diagnosis of NS.

**Figure 2:**
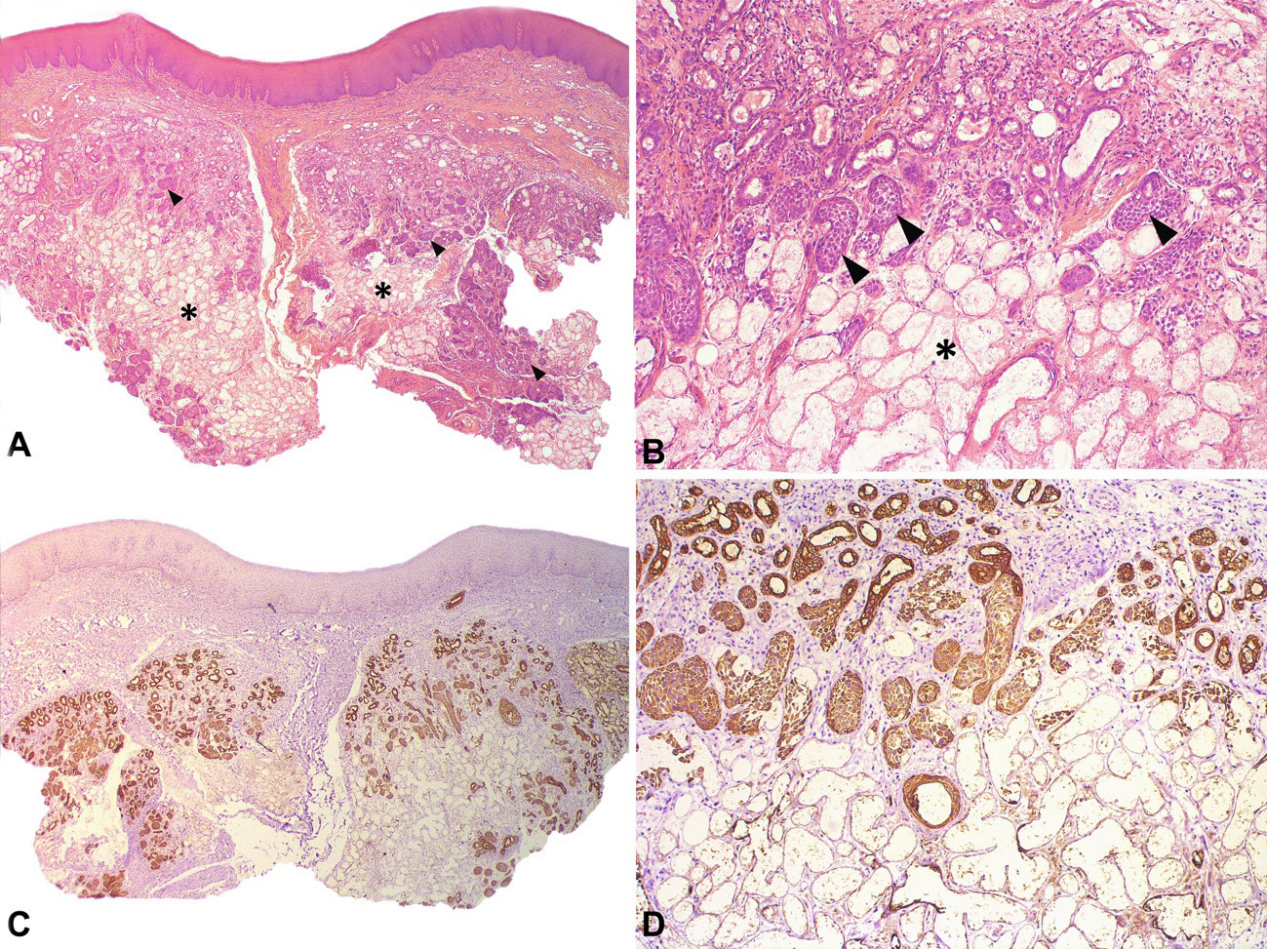
Photomicrographs of the biopsy showing in **A** – large areas of acinar necrosis (*) associated with foci of squamous metaplasia (arrowhead) beneath a normal surface epithelium (H&E, 25X); **B** – Ductal squamous metaplasia and large areas of acinar necrosis were present, preserving the lobular glandular architecture (H&E, 25X); **C** – ductal epithelium showed strong and diffuse positivity to CK7 (100X); **D** – loss of immunopositivity for CK7 in the necrotic areas (100X).

**Figure 3:**
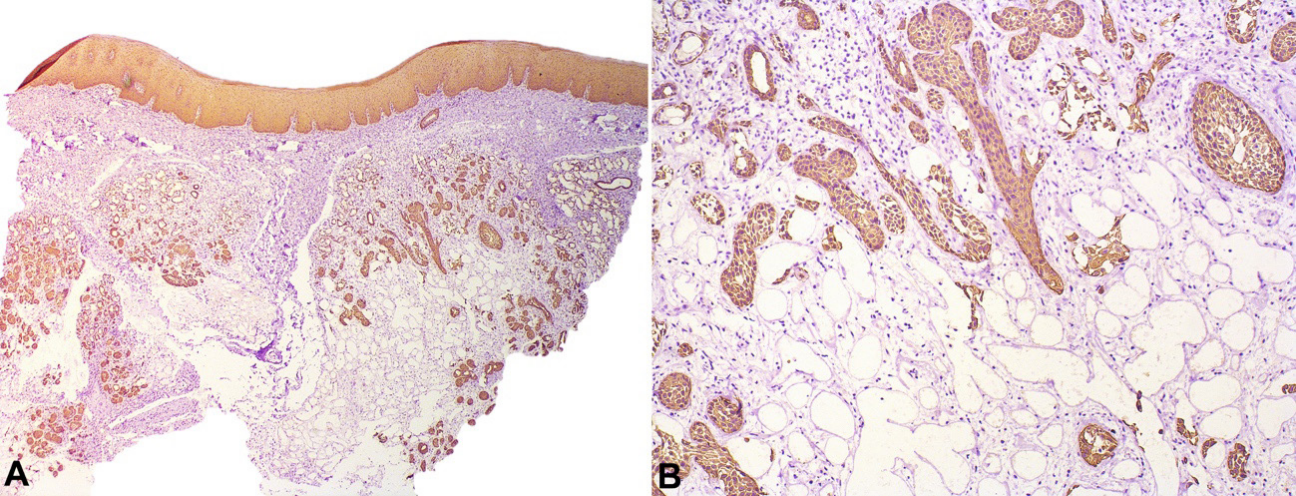
Photomicrographs of the biopsy showing in **A** and **B** – positivity for CK 14 in the areas of squamous metaplasia (**A** – 25X, and **B** – 100X).

**Figure 4:**
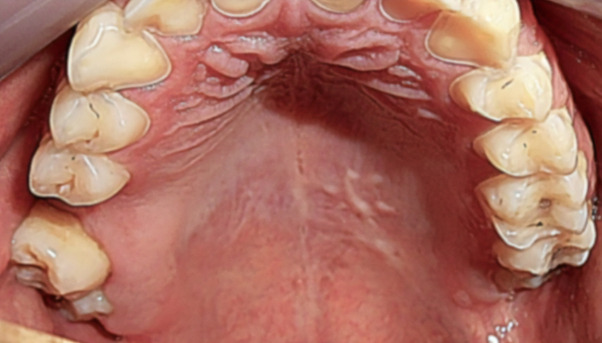
Final clinical feature showing total healing after a few days of the biopsy procedure.

As NS is a self-limiting condition, the patient received supportive treatment focused on controlling pain with use of an analgesic every 6 hours for 2 days. After the biopsy procedure, the lesion evolved toward complete resolution, reaching it in a few days (Figure 4).

## DISCUSSION

NS is a rare condition, with just over 200 cases reported in the English language literature.^2^ It represents less than 1% of all biopsies carried out in the mouth.^1^ First described in 1973 by Abrams and colleagues,^13^ this condition can affect any site that contains salivary glands. The majority of cases occur inside the mouth, most often in the soft or hard palate where minor salivary glands are found. Although less frequent, major salivary glands may also be affected.^14^ ^,^ ^15^ As presented in the current case, NS primarily affects middle-aged adult male patients. Clinically, NS often presents as a deep, necrotic ulcer surrounded by an erythematous halo. NS usually occurs lateral to the midline, but larger lesions can cross the midline and NS can occasionally be bilateral.^1^ ^,^ ^5^ ^,^ ^9^ ^,^ ^12^ If the patient reports any history of trauma, local anesthesia, poor-fitting prosthesis or any other source of tissue ischemia in association with the clinical features, a diagnosis of NS should be strongly considered.

The diagnosis becomes a challenge in non-ulcerated NS, as reported in the current case. When the palate presents swelling or a nodular lesion, it is essential to rule out the possibility of salivary gland tumors, including malignant ones. The presence of pain and color alteration (blue and purple, specifically) have been described as independent predicting factors of malignancy.^4^ The early aspect of NS (before central ulceration is evident) may mimic the clinical aspect of these tumors, as pain, for instance, is a common complaint. Nevertheless, in most cases, salivary gland tumors do not present a short evolution time like NS.^1^ Table 1 presents a summary of 10 previous similar cases of non-ulcerated NS reported in the English language literature over the last 20 years (2000–2020) and the current report. These cases represent examples of patients who sought care, presenting with swelling, nodules or areas of color change at the time of the appointment. Different to the case reported here, some of them evolved to an ulcer a few days after the first visit. This may be explained, in part, by some clinicians opting to wait and observe the lesion for a couple of days before performing a biopsy. Considering only the seven patients with unilateral lesions, pain was present in four patients and color alteration in four cases, as well. In two patients, including the current case, pain and color alteration occurred simultaneously, making it even more challenging to clinically differentiate them from a malignant salivary gland tumor. Eating disorders were the most cited triggering factor (four cases), and substance abuse was reported in two patients, including the current case. Table 1 also shows that, in general, the time of evolution of SN is shorter than that of salivary gland tumors, typically resolving in less than 30 days.

**Table 1:** Clinical features of 11 cases of non-ulcerated necrotizing sialometaplasia

Reference	Age/Gender	Clinical features	Anatomic site	Side	Possible triggering factor	Evolution time (days)	Symptoms	Color alteration
Keogh et al.^5^	30/F	Swelling	S/H P	Bilateral	Other	12	Numbness	Erythematous
Femopase et al.^6^	17/F	Nodule	HP	Left	ED/vomiting	90	Tenderness	Erythematous/purple
Oliveira et al.^7^	25/F	Nodule	HP	Right	Not reported	21	Pain	No
Kaplan et al.^1^	29/F	NM	SP	Right	SA	ND	Pain	ND
Kaplan et al.^1^	33/F	NM	SP	Bilateral	ED/vomiting	3	Numbness + pain	ND
Kaushal et al.^10^	40/F	Swelling	HP	Left	anesthesia	56	Tenderness	No
Janner et al.^9^	22/F	Nodule	HP	Bilateral	ED/vomiting/SA	15	Pain	Erythematous
Arpaci et al.^8^	58/M	Swelling	HP	Left	Not reported	21	ND	Blue/Purple
Shetty et al.^11^	35/F	Swelling	HP	Right	Not reported	15	Pain	Blue/Purple
Rushinek et al.^12^	49/F	Swelling	HP	Bilateral	ED/vomiting	15	Numbness + pain	ND
our case	32/M	EM	HP	Right	SA	30	Pain	Erythematous

ED= eating disorders; EM = ELEVATED MUCOSA; HP = HARD PALATE; ND= not described; NM=normal mucosa; SA= substance abuse, SP=soft palate; S/P=soft/hard.

In these cases, biopsy is crucial to elucidate the correct diagnosis, although similarities may also occur in histopathological examination. NS usually presents squamous metaplasia of the salivary ducts, which could be dominant and may present pseudoepitheliomatous hyperplasia of the overlying epithelium, acinar necrosis and mucin shedding. These histopathological features generate a pattern that resemble malignant tumors such as mucoepidermoid carcinoma or even squamous cell carcinoma. However, when a detailed examination is performed, it is possible to observe an intact lobular architecture as well as squamous islands with placid morphology, indicating a benign origin.^1^ ^,^ ^2^ ^,^ ^13^ Immunohistochemistry can help to confirm the diagnosis of NS in some situations. CK7 has been shown to be an interesting marker to differentiate NS from mucoepidermoid carcinoma and squamous cell carcinoma. While NS presents moderate staining for this marker, mucoepidermoid carcinoma presents extensive staining, especially in mucous cells and epidermoid areas, and squamous cell carcinoma may present focal or negative staining.^16^ ^,^ ^17^

Furthermore, the identification of a triggering factor can also help to elucidate the origin of this entity. An ischemic event is recognized as the main cause of NS, and such ischemia can occur several ways, including local trauma, poor-fitting prosthesis and local anesthesia.^1^ ^,^ ^2^ Other causes include abuse of certain substances including tobacco, alcohol and vasoconstricting drugs, such as cocaine, among others.^1^ ^,^ ^18^ ^,^ ^19^ Additionally, patients presenting eating disorders, especially those who induce vomiting, have been reported to develop NS, and patients who present certain systemic diseases, such as diabetes mellitus, can also presents with this condition.^1^ ^,^ ^6^ ^,^ ^9^ ^,^ ^12^ ^,^ ^18^ Rarely, NS can be associated with a tumor growth, as the pressure of the tumor could lead to tissue ischemia.^2^ In the case presented herein, the patient reported cocaine abuse; however, he did not specify whether he snorts it or rubs it on soft tissue like the gingiva or hard palate. The patient also reported alcohol and tobacco use, but it is difficult to directly associate those factors with the appearance of the lesion.

In summary, NS is a benign and self-limiting condition that usually presents clinically as an ulcerated lesion. Occasionally, however, NS may manifest as a non-ulcerated aspect. Therefore, this report aimed to highlight a case of early-stage and non-ulcerated NS to alert clinicians to consider this clinical spectrum of NS as a differential diagnosis of minor salivary gland tumors.
